# Physical reparative treatment in reptiles

**DOI:** 10.1186/1746-6148-9-39

**Published:** 2013-02-26

**Authors:** Salvatore Rinaldi, Maddalena Iannaccone, Gian Enrico Magi, Emanuela Costantini, Alessandro Castagna, Eraldo Sanna Passino, Margherita Maioli, Vania Fontani

**Affiliations:** 1 Department of Regenerative Medicine, Rinaldi Fontani Institute, Viale Belfiore 43, Florence 50144, Italy; 2 Il mondo degli animali esotici, Via S. Martino 67/r, Genoa 16131, Italy; 3 School of Veterinary Medical Sciences, University of Camerino, Via Circonvallazione 93/95, Matelica (MC) 62024, Italy; 4 Society of Neuro Psycho Physical Optimization, and REAC technology, Viale Belfiore 43, Florence 50144, Italy; 5 School of Veterinary Medical Sciences, University of Sassari, Sassari, Italy; 6 Department of Biomedical Sciences, University of Sassari, Sassari, Italy; 7 Laboratory of Molecular Biology and Stem Cell Engineering, National Institute of Biostructures and Biosystems, Bologna, Italy

**Keywords:** Tissue repair, Tissue optimization, Tortoise, Turtle, Radio electric asymmetric conveyer

## Abstract

**Background:**

The tissue growth necessary to achieve a complete or partial restitution ad integrum as a result of injury to soft tissue and/or hard times in reptiles is variable and often needs long time in relation to the species, to the habitat and to their intrinsic physiological characteristics. The purpose of this work was to see if the tissue optimization (TO) treatment with radio electric asymmetric conveyer (REAC) provided good results in these animals and whether its use translates into reduced time of tissue repair. This paper describes preliminary results with in promoting the tissue repair in reptiles.

**Cases presentation:**

A 5 year old male Testudo graeca (Leo) and Trachemys scripta scripta (Mir) and a 15 year old female Testudo hermanni (Juta) were evaluated because of soft tissue injuries. A female 25 year old Trachemys scripta elegans (Ice), a female 2.5 year old Trachemys scripta scripta (Penelope) as well as a 50 year old male Testudo graeca (Margherito) were evaluated because of wounds of the carapace. Following debridement and traditional therapies, Leo, Penelope and Margherito were exposed to the radio electric asymmetric conveyer (REAC) device, with a specific treatment protocol, named tissue optimization-basic (TO-B). Also Ice and Mir were subjected to REAC treatment after wounds debridement. Juta was treated only with REAC treatment.

Complete wound healing was evident after 17 days for Leo, 7 days for Penelope, 27 days for Mir, 78 days for Ice and after 14 days for Margherito. Juta showed a considerable tissue activation in 2 days and complete wound healing in 5 days.

**Conclusion:**

Our findings suggest that REAC TO-B treatment may provide advantages over other traditional methods after complete wound healing in Leo, and also suitable healing in the other patients. Then REAC device with its specific treatment TO-B protocol, which induces tissue repair without causing severe stress to the patient, could be a potential therapy for tissue damage healing in reptiles. Further studies still need to be conducted to support our observations.

## Background

Among the various methods used to treat wounds [1,2] such as beds, compression, hydrotherapy, therapeutic ultrasound, negative pressure therapy, laser therapy, an increase in the rate of tissue repair has been obtained by other authors using electrical stimulation [3-5] and magnetic fields, both in humans and in animals [6-8]. More recently, an innovative technology, radio electric asymmetric conveyer (REAC), with its specifics treatment protocol defined with the general name of tissue optimization (TO) has proven efficacy in inducing cell pluripotency and differentiation in different cell lines, including embryonic stem cells [9] and human skin-derived fibroblasts, [10] representing a new tool for improving tissue regeneration. REAC TO has proven efficacy also in ameliorating tissues healing [11-15] and was also successfully used for the treatment of post-traumatic injury and surgical wounds both in humans and in animals [11-15]. Recent studies have demonstrated the efficacy of REAC TO also in the osteoarthritic chondrocytes repair [16]. In the present clinical study we investigated if a protocol of this innovative treatment named REAC TO-base (TO-B) was able to ameliorate tissue repair in a Testudo graeca and a Trachemys scripta scripta with severe traumatic injuries which till this moment did not show a significant improvement of lesions with traditional treatments that in one case were applied for a long period. The REAC TO-B treatments were applied also in a Trachemys scripta elegans and in another Trachemys scripta scripta that did not received other traditional treatments. The purpose of this case report is to describe our observations using tissue optimization-basic (TO-B) treatment with a radio electric asymmetric conveyer (REAC) device and how this may translate into reduced time of tissue repair in this type of animal.

## Case presentation

**Figure 1:**
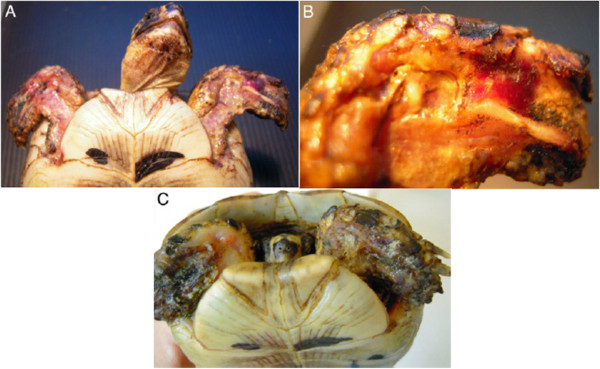
**A) Day One: A young *Testudo graeca*, Leo, was found with these lesions produced by rat bites. B**) Detail of the severe injury localized dorsal left front limb. Note the considerable loss of substance and humeral-radioulnar joint and bone exposure. **C**) The lesions after 2 months and after repeated applications of antibiotics, disinfectants and healing cream and before to start radio electric asymmetric tissue optimization treatment.

**Figure 2:**
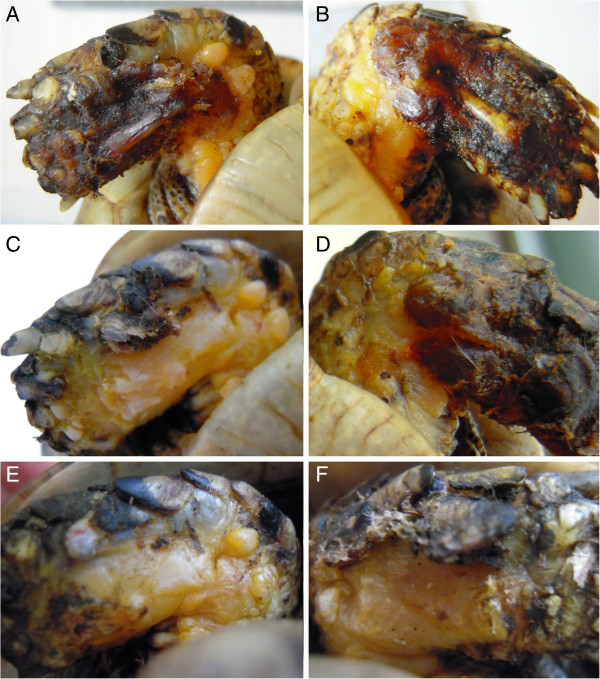
**A-B) Lesions of front limbs (Leo) after 4 treatments of REAC-TO and 2 days after the Figure**1**C. C**-**D**) Lesions of front limbs after 8 treatments of REAC-TO and 5 days after the Figure 1C. **E**-**F**) Lesions of front limbs after 12 REAC-TO treatments and 17 days after the Figure 1C.

**Figure 3:**
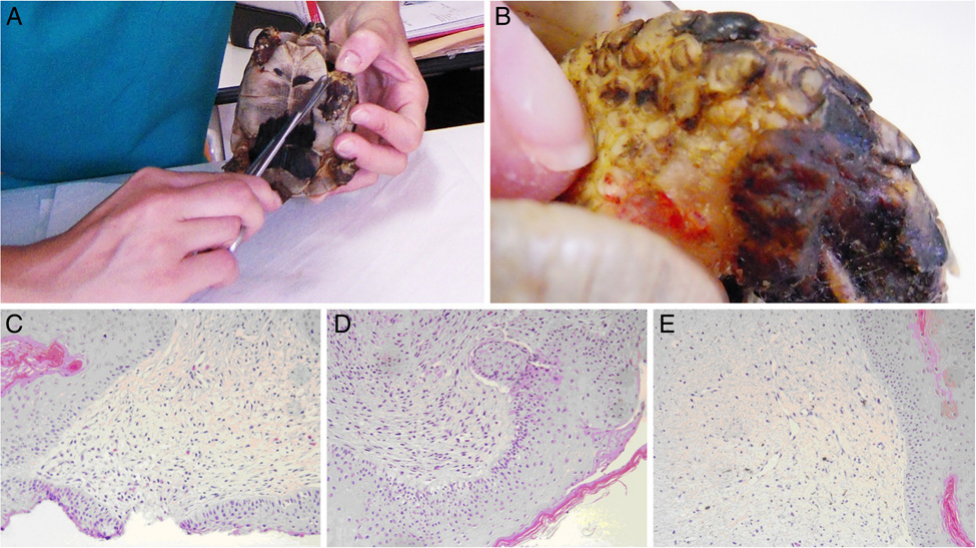
**A-B) One of the biopsies taken during REAC-TO treatment. C**) Skin. Area of moderate cutaneous excoriation. The underlying dermis present mixed inflammatory cells and some eosinophilic granular cells (HE, 10X). **D**) Skin 3 day post-treatment. The upper dermis present numerous fibroblast and abundant fibrous matrix (HE, 10X) **E**) Skin 7 day post-treatment. Fibroplasias in the upper dermis (HE, 10X). Serial 4 μm sections were stained with haematoxylin and eosin (HE).

**Figure 4:**
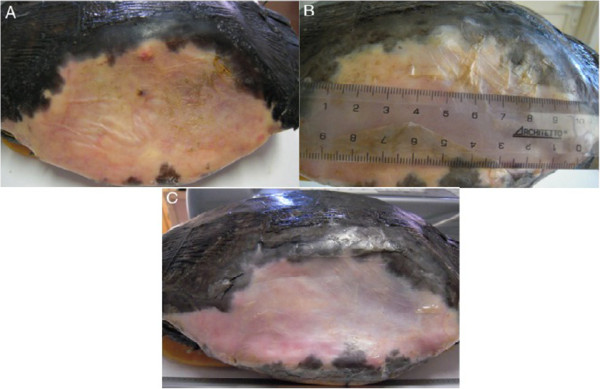
**A) Adult female *Trachemys scripta elegans, *Ice, with serious injury by freezing and considerable loss of shell.** Detail of the lesion before REAC-TO treatment. **B**) Detail of the same lesion after 2.5 REAC-TO treatment cycle corresponding to 2 months. **C**) Detail of the same lesion 78 days after the first REAC-TO treatment.

**Figure 5:**
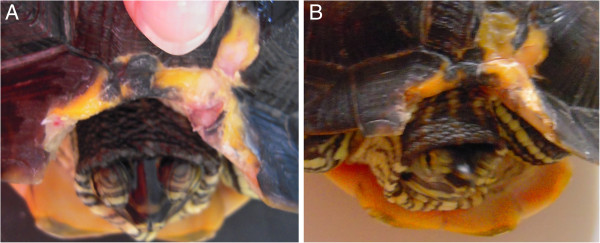
**A) *Trachemys scripta scripta*, 2.5 years old infected wound of the shell, treated with REAC-TO. B**) The same animal after 18 REAC-TO sessions, 7 days later.

**Figure 6:**
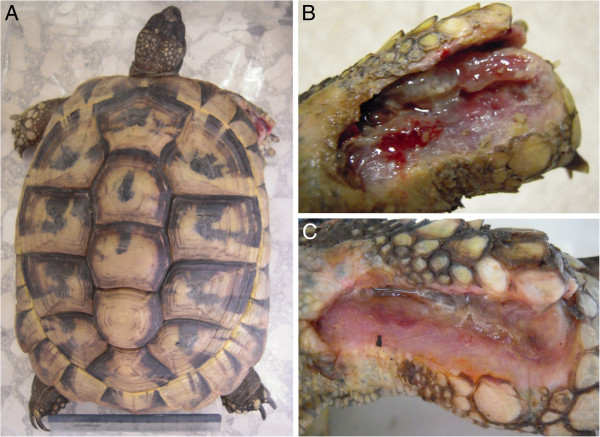
**A) Adult female *Testudo Hermanni, *Juta, with a lesion of right front limb. B**) Detail of the lesion before radio electric asymmetric tissue optimization treatment. **C**) Detail of the same lesion after 12 cycles of radio electric asymmetric tissue optimization treatment and 2 days later.

**Figure 7:**
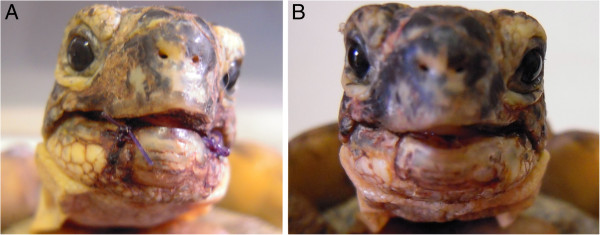
**A) Adult male *Testudo graeca, *Margherito, with a fractured jaw surgically repositioned. B**) The same animal 2 weeks later after 12 cicles of of radio electric asymmetric tissue optimization treatment.

**Figure 8:**
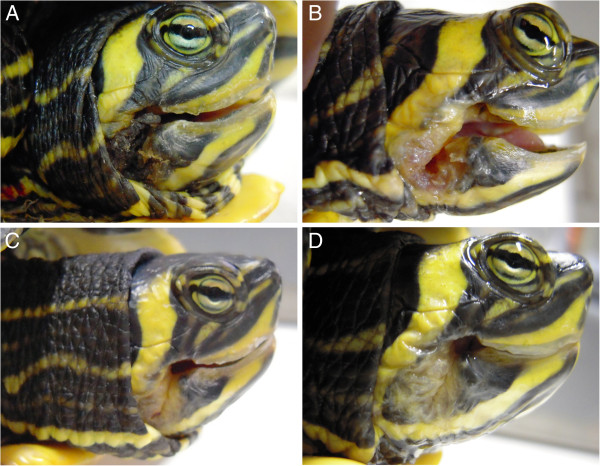
**A) Trachemys scripta scripta, 5 years old affected by necrosis of the mouth, caused by a hook. B**) After 12 REAC-TO sessions, 15 days later. **C**) After 18 REAC-TO sessions, 20 days later. **D**) 27 days after the first REAC-TO treatment.

A 5 years old male Testudo graeca (Leo), of 500 gr weight was brought to our attention in May with skin and muscle injuries localized in particular in the dorsal front limbs caused by rat bites 2 days before (Figure 1A). The tortoise, which had come out of hibernation in March, live in a garden near a landfill. These severe injuries, localized in particular in the dorsal left front limb, (Figure 1B) showed a considerable loss of substance and humeral-radioulnar joint and bone exposure. The wounds were lightly contaminated with soil and showed a small tissue necrosis. After debridement of all necrotic and non-viable tissues, a local disinfection with a mixed solution of sodium chloride, hydrogen peroxide and iodopovidone for 2 weeks, enrofloxacin IM 5 mg/kg once for day (Baytril 2, 5%, Bayer) for 2 weeks, and ceftazidime IM 20 mg/kg once for day (Glazidim, GlaxoSmithKline) for 5 weeks were administered; moreover chloramphenicol and collagenase based cream (Iruxol cream 1%, Smith+Nephew) for 3 weeks after systemic antibiotics was locally dispensed. During this period the reptile was kept in an acclimatized terrarium with the temperature ranging from 24 to 32°C, and artificial sun light (UVB 10% for 6 hours for day). After 60 days, because no significant improvements in wound repair were observed, (Figure 1C) we decided to expose the reptile to REAC TO-B treatment. Therefore all the other therapies were stopped one day before the beginning of this innovative therapy. The animal was submitted to 12 sessions of REAC TO-B along 17 days. This treatment required the use of anesthesia, only during the first 4 sessions (alphaxalone IM 20 mg/Kg), afterword anesthesia was no more necessary, because the animal was calm, and during the last session it was asleep. During the time of treatment the tortoise eats regularly its usual diet. After 12 sessions of REAC TO-B tissue growth was evident in injuries localized in both front limbs (Figure 2 A-B-C-D-E-F). Considering the tissue damages occurring during the first 2 months of traditional treatments, after 17 days of REAC TO-B treatment there was an evident increase in recovery of both legs’ lesions. This tissue recovery, growth and remodeling, was confirmed by histological analysis obtained evaluating cutaneous biopsies taken at the level of the lesions, before (T0), 3, 7 (Figure 3 A-B-C-D-E) and 14 days after the first REAC TO-B treatment. Before REAC TO-B treatment underlying dermis of moderate cutaneous excoriation appeared infiltrated by mixed mononuclear inflammatory cells and some eosinophilic granular cells. Noteworthy just three days after the first REAC TO-B session the upper dermis presented numerous fibroblasts surrounded by extracellular matrix and few mononuclear inflammatory cells, whereas the epidermis appeared hyperplasic. Four days later skin biopsy showed mature connective tissue and tissue remodeling. After 17 days there was a complete healing. Considering the success obtained in the case of tortoise Leo we treated with REAC TO-B therapy also other turtles which presented lesions in soft tissue and in the carapace. In particular: a Trachemys scripta elegans (female, 25 years old, Ice) which exhibited a shell wound due to partial freezing (Figure 4), a Trachemys scripta scripta (female 2.5 years old, Penelope), with a small infected wound of the shell (Figure 5), a Testudo hermanni (female 15 years old, Juta) with a traumatic lesion of right front limb (Figure 6), a Testudo Graeca (male 50 years old, Margherito) with a jaw fracture (Figure 7) caused by a dog bite as well as a Trachemys scripta scripta (male 5 years old, Mir) with a necrosis of the mouth, caused by a hook (Figure 8). Margherito has been subjected to surgery and was treated with ceftazidime IM 20 mg/kg once (Glazidim, GlaxoSmithKline) for 2 weeks before REAC-TO treatment. Ice and Mir exposed to REAC TO-B were not previously treated with other pharmacological therapies except wounds debridement, while Penelope, after debridement, was subjected to local disinfection of the infected shell with a mixed solution of sodium chloride and iodopovidone for four days. Juta didn’t receive any treatment before REAC-TO. Before starting REAC TO-B treatment all the previously administered therapies were stopped.

**Figure 9:**
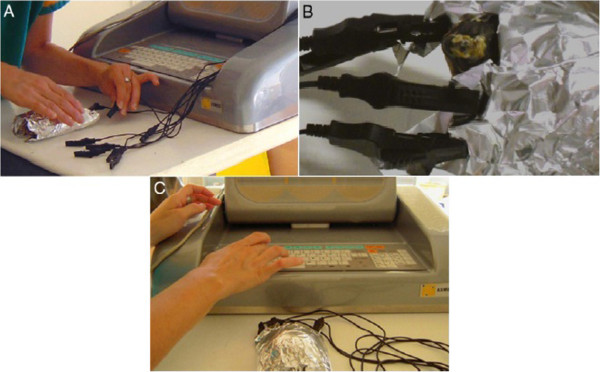
**A) Patient preparation (Leo) for REAC-TO treatment. B**) Detail of electrodes placement during the REAC-TO. **C**) Equipment for REAC-TO treatment.

The REAC is an innovative-patented technology (WO2002004069) for bio-stimulation and/or bio-enhancement techniques that induces weak radio-electric currents in the tissues, to induce a cell reprogramming activity. The model used in this study (ASMED, Florence, Italy) is specific for regenerative treatments. The REAC-TO protocol consisted of 100 radio frequency bursts, each of 2.4 GHz for 0.5 seconds, with a specific absorption rate of 7 μW/kg, spaced with 4.5-second pauses, applied to the skin by a special laminar aluminum electrode (Figure 7). Each therapy session lasted about 10 minutes, with 18 sessions constituting a REAC TO-B treatment cycle. A REAC model (ASMED, Florence, Italy), was used in this study. During therapy the patients were completely wrapped in a special laminar aluminum electrode, specific for REAC TO-B treatments, in a way that the ends of the aluminum electrode were not touching each other. The conveyors electrodes were placed in this way: 4 in the cranial part of the aluminum foil and 4 in the caudal part (Figure 9). The distribution of daily sessions was organized according to the eventual administration of anesthesia [17] and to the response of the animal to handling: when the patient was calm and showed no signs of discomfort, more sessions for day were performed for a maximum of 6 sessions for day (the animals were more calm if we covered them with a dark cloth).

As previously described for Leo all the other turtles were exposed to 12 or more sessions of REAC TO-B treatment, by the aid of anesthesia or not along different period of time. The ameliorations of the shell of the animal after REAC TO-B treatments are reported in Figure 5. In particular the case of the animal showing a loss of the shell (Ice) was particularly evident because of the possibility of verifying the scarring and hardening of the tissue underlying the lesion. In fact, after 2.5 REAC TO-B treatment cycle, corresponding to 60 days, the animal showed a marked narrowing of the area of exposure of the peritoneal membrane. This resulted in a significant reduction in visibility of raising and lowering related to breathing, that were highly visible before treatment. Penelope (Figure 5) began REAC TO-B treatment after local disinfection was applied for four days. For this reason 18 sessions of REAC TO-B, corresponding to 7 days were enough to see evident wound healing (Figure 5B).

After 18 sessions of REAC TO-B, corresponding to 20 days, Mir showed wound reepithelization and necrosis disappearance, and a complete healing over the next 7 days (Figure 8D).

All the animals were monitored after REAC TO-B treatments; 2 of them for 18 months and 4 for 12 months and we didn’t see any long term disorder or problem.

Macroscopic and histological results underline a significant tissue repair based on clinical observation in Leo and other reptiles. Thereby wound healing also in tortoises proceeds by a process of granulation, epithelialization and wound contraction, [18,19] which generally takes several weeks to heal, and has been shown to be temperature dependent.

In the present work we evidenced that *Testudo graeca* Leo, and 3 turtles with different injuries of soft and hard tissues treated with REAC-TO showed an evident amelioration of healing wounds. It is known that the reptilian epidermis is composed of a beta-keratin layer, the mesos layer and the alpha-keratin layer, [20] which is supported by the underlying stratum germinativum, whereas the chelonian shell is composed of a thick epithelium and contains layers of keratin [20]. The deeper dermis, derived from embryonic mesoderm contains connective tissues, vascular tissues, sensory structures and dermal bone (osteodermis). We have previously demonstrated that REAC TO-B treatment induces cell proliferation and differentiation toward different lineages in vitro [9,10]. In particular REAC-TO was found to have the ability to modulate the expression of genes and proteins involved in the differentiation of embryonic mouse cells in vitro [9]. Moreover we recently observed that REAC-TO influenced the plasticity and differentiation capability of human skin derived fibroblasts toward different cellular lineages, [10] thus further demonstrating the modulatory effect of this device on cell fate and tissue regeneration. Therefore we can argue that the amelioration of wound healing and bone fracture of reptiles observed here may be due to a proliferation of the germinativum stratum of the epidermis and of the embryonic mesoderm responsible for the formation of connective tissues, vascular tissues, sensory structures and osteodermis. Osteodermis is composed of a mixture of spongy and compact bone and, in tortoises, is fused with the ribcage and spine, expanding to form the plates of bone that make up the chelonian carapace and plastron [20-22]. Our patients were subjected to different sessions of REAC-TO treatment, ranging from 12 (Leo) to 42 (Ice), considering the site and seriousness of injury. Therefore as supposed the number of REAC-TO sessions was higher in the patient exhibiting lesions of shell (Ice). The REAC-TO treatment, applied after the complete debridement of the necrotic tissues, did not cause a severe stress to patient and required only few anesthetic treatments (Leo). The positive action of REAC-TO treatment is further inferred by clinical results obtained by us in healthy human subjects, improving circulation, hydration, and the tropism of facial skin [13].

Since REAC-TO is able to activate the process of tissue repair by inducing cellular organization and a vascular network, activating and accelerating tissue recovery may represent a powerful approach that could pave new ways in veterinary and human healing, besides other physical and chemical treatments [23,24].

## Conclusion

In conclusion our data suggest that REAC TO-B could be a new tool for treating injuries in reptiles. More detailed studies are needed in order to confirm these results.

### Ethics

This study has been approved by Veterinary control officers of Animal protection in experimental and clinical studies made in University of Sassari, Italy (directive C.E.E. n.86/609). We have obtained the consent to treatment, from each owner, before starting the study.

## Competing interest

Salvatore Rinaldi and Vania Fontani are the inventors of the Radio Electric Asymmetric Conveyer.

## Authors’ contributions

SR and VF invented REAC, developed the experimental design, and wrote the manuscript. MI performed the experiments and wrote the manuscript. EC and AC, performed the experiments. GEM performed histological analysis. MM and ESP designed/supervised the project and wrote the manuscript. All authors read and approved the final manuscript.
